# Supplementary material for: Utilization of diabetes self-management program among breast, prostate, and colorectal cancer survivors: Using 2006–2019 Texas Medicare data
Source: PLoS One. 2023 Jul 27;18(7):e0289268. doi: 10.1371/journal.pone.0289268 (PMC10374119; doi:10.1371/journal.pone.0289268)
Supplement: S1 File — (DOCX) [file pone.0289268.s001.docx]

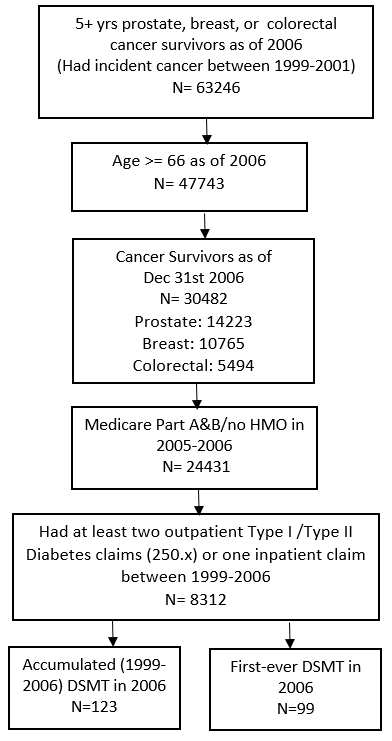


**S1 Fig.** Flow Chart to Draw Eligible Cohort in 2006

**S1 Table. Model Predicting the First DSMT Use among Prostate Cancer Survivors (Male Only)**

| **Variables** | **First DSMT Use Rate** | **OR*** | **95% CI** | | ***p*-value** |
| --- | --- | --- | --- | --- | --- |
| **Year** |  |  |  |  |  |
| 2006 (Referent) |  | 1.00 | - | - | - |
| 2007 |  | 1.11 | 0.74 | 1.66 | 0.6246 |
| 2008 |  | 1.10 | 0.75 | 1.62 | 0.6214 |
| 2009 |  | 0.74 | 0.49 | 1.10 | 0.1377 |
| 2010 |  | 0.69 | 0.46 | 1.02 | 0.0639 |
| 2011 |  | 0.70 | 0.48 | 1.04 | 0.0745 |
| 2012 |  | 0.57 | 0.39 | 0.85 | 0.0058 |
| 2013 |  | 0.51 | 0.34 | 0.76 | 0.0009 |
| 2014 |  | 0.85 | 0.59 | 1.23 | 0.3895 |
| 2015 |  | 2.42 | 1.73 | 3.39 | <.0001 |
| 2016 |  | 1.18 | 0.83 | 1.69 | 0.357 |
| 2017 |  | 0.98 | 0.68 | 1.40 | 0.9008 |
| 2018 |  | 0.82 | 0.57 | 1.19 | 0.3046 |
| 2019 |  | 0.95 | 0.65 | 1.37 | 0.7643 |
| **Age Group** |  |  |  |  |  |
| 66-74 (Referent) | 686/57709 | 1.00 | - | - | - |
| 75-84 | 705/87543 | 0.69 | 0.62 | 0.78 | <.0001 |
| 85+ | 148/29309 | 0.41 | 0.34 | 0.49 | <.0001 |
| **Race/Ethnicity** |  |  |  |  |  |
| Non-Hispanic White (Referent) | 1013/122765 | 1.00 | - | - | - |
| Non-Hispanic Black | 173/19077 | 0.96 | 0.81 | 1.14 | 0.6723 |
| Hispanic | 327/29529 | 1.17 | 1.01 | 1.36 | 0.0340 |
| Other | 26/3190 | 0.88 | 0.58 | 1.34 | 0.5535 |
| **Newly Diagnosed Diabetes** | 231/7413 | 4.70 | 4.03 | 5.47 | <.0001 |
| **Diabetes with Complications (Referent: No complications)** | 978/95290 | 1.74 | 1.56 | 1.94 | <.0001 |
| **Medicare Original Entitlement: Disabled (Referent: Not disabled)** | 120/13279 | 0.88 | 0.72 | 1.07 | 0.2095 |
| **Dual Eligibility (Referent: No dual eligibility)** | 229/18203 | 1.54 | 1.30 | 1.83 | <.0001 |
| **Quartiles of Percent Living in Regions with < 12 Years Education** |  |  |  |  |  |
| [0-11] (Referent) (Very good education) | 427/49441 | 1.00 | - | - | - |
| [12-20] (Good education) | 323/39515 | 0.94 | 0.81 | 1.08 | 0.3885 |
| [21-29] (Poor education) | 315/37240 | 0.92 | 0.79 | 1.09 | 0.3389 |
| [30-100] (Very poor education) | 364/37713 | 1.00 | 0.85 | 1.16 | 0.9547 |
| **Metropolitan Areas (Referent: Non-Metro)** | 1218/142449 | 0.83 | 0.72 | 0.96 | 0.0096 |
| **Elixhauser comorbidity index** |  |  |  |  |  |
| [0-1] (Referent) | 418/42562 | 1.00 | - | - | - |
| [2-3] | 583/61913 | 1.00 | 0.88 | 1.14 | 0.9754 |
| [>3] | 538/70086 | 0.84 | 0.73 | 0.96 | 0.0118 |

*OR= Odds Ratio.

**S2 Table. Model Predicting the First DSMT Use among Breast Cancer Survivors (Female Only)**

| **Variables** | **First DSMT Use Rate** | **OR*** | **95% CI** | | ***p*-value** |
| --- | --- | --- | --- | --- | --- |
| **Year** |  |  |  |  |  |
| 2006 (Referent) |  | 1.00 | - | - | - |
| 2007 |  | 0.63 | 0.40 | 0.99 | 0.0456 |
| 2008 |  | 0.73 | 0.48 | 1.10 | 0.1343 |
| 2009 |  | 0.74 | 0.49 | 1.10 | 0.135 |
| 2010 |  | 0.65 | 0.43 | 0.96 | 0.0323 |
| 2011 |  | 0.55 | 0.37 | 0.82 | 0.0035 |
| 2012 |  | 0.67 | 0.46 | 0.98 | 0.0413 |
| 2013 |  | 0.59 | 0.40 | 0.86 | 0.0069 |
| 2014 |  | 0.78 | 0.54 | 1.13 | 0.1929 |
| 2015 |  | 1.79 | 1.27 | 2.51 | 0.0008 |
| 2016 |  | 0.87 | 0.61 | 1.25 | 0.4569 |
| 2017 |  | 1.01 | 0.71 | 1.43 | 0.9684 |
| 2018 |  | 0.77 | 0.54 | 1.11 | 0.1677 |
| 2019 |  | 0.75 | 0.52 | 1.08 | 0.1195 |
| **Age Group** |  |  |  |  |  |
| 66-74 (Referent) | 817/56286 | 1.00 | - | - | - |
| 75-84 | 533/63768 | 0.59 | 0.53 | 0.66 | <.0001 |
| 85+ | 131/29200 | 0.34 | 0.28 | 0.41 | <.0001 |
| **Race/Ethnicity** |  |  |  |  |  |
| Non-Hispanic White (Referent) | 969/105338 | 1.00 | - | - | - |
| Non-Hispanic Black | 158/14464 | 1.08 | 0.91 | 1.29 | 0.3857 |
| Hispanic | 321/26408 | 1.19 | 1.03 | 1.37 | 0.0196 |
| Other | 33/3044 | 1.14 | 0.81 | 1.61 | 0.4402 |
| **Newly Diagnosed Diabetes** | 222/5852 | 5.12 | 4.36 | 6.02 | <.0001 |
| **Diabetes with Complications (Referent: No complications)** | 938/80822 | 1.78 | 1.59 | 2.00 | <.0001 |
| **Medicare Original Entitlement: Disabled (Referent: Not disabled)** | 112/10885 | 0.87 | 0.71 | 1.07 | 0.1973 |
| **Dual Eligibility (Referent: No dual eligibility)** | 274/24741 | 1.07 | 0.92 | 1.24 | 0.3741 |
| **Quartiles of Percent Living in Regions with < 12 Years Education** |  |  |  |  |  |
| [0-11] (Referent) (Very good education) | 363/38317 | 1.00 | - | - | - |
| [12-20] (Good education) | 331/35620 | 0.98 | 0.85 | 1.14 | 0.8052 |
| [21-29] (Poor education) | 337/33852 | 1.02 | 0.87 | 1.19 | 0.8137 |
| [30-100] (Very poor education) | 359/33865 | 1.04 | 0.89 | 1.21 | 0.6394 |
| **Metropolitan Areas (Referent: Non-Metro)** | 1177/123270 | 0.78 | 0.68 | 0.90 | 0.0005 |
| **Elixhauser comorbidity index** |  |  |  |  |  |
| [0-1] (Referent) | 301/25704 | 1.00 | - | - | - |
| [2-3] | 580/54244 | 0.99 | 0.85 | 1.14 | 0.8437 |
| [>3] | 600/69306 | 0.84 | 0.73 | 0.97 | 0.0212 |

*OR= Odds Ratio.

**S3 Table. Model Predicting the First DSMT Use among Colorectal Cancer Survivors (Both Genders)**

| **Variables** | **First DSMT Use Rate** | **OR*** | **95% CI** | | ***p*-value** |
| --- | --- | --- | --- | --- | --- |
| **Year** |  |  |  |  |  |
| 2006 (Referent) |  | 1.00 | - | - | - |
| 2007 |  | 1.18 | 0.60 | 2.32 | 0.6411 |
| 2008 |  | 1.02 | 0.53 | 1.99 | 0.9429 |
| 2009 |  | 0.82 | 0.42 | 1.61 | 0.5704 |
| 2010 |  | 0.68 | 0.35 | 1.33 | 0.2644 |
| 2011 |  | 0.48 | 0.24 | 0.97 | 0.0403 |
| 2012 |  | 0.78 | 0.41 | 1.47 | 0.4422 |
| 2013 |  | 0.41 | 0.20 | 0.84 | 0.0145 |
| 2014 |  | 1.16 | 0.64 | 2.11 | 0.6269 |
| 2015 |  | 3.23 | 1.85 | 5.64 | <.0001 |
| 2016 |  | 1.44 | 0.80 | 2.59 | 0.2181 |
| 2017 |  | 1.06 | 0.58 | 1.93 | 0.8542 |
| 2018 |  | 1.32 | 0.73 | 2.38 | 0.3554 |
| 2019 |  | 1.10 | 0.60 | 2.00 | 0.758 |
| **Sex** |  |  |  |  |  |
| Male (Referent) | 302/35234 | 1.00 | - | - | - |
| Female | 309/33498 | 1.15 | 0.98 | 1.36 | 0.093 |
| **Age Group** |  |  |  |  |  |
| 66-74 (Referent) | 330/21856 | 1.00 | - | - | - |
| 75-84 | 199/30397 | 0.45 | 0.38 | 0.55 | <.0001 |
| 85+ | 82/16479 | 0.32 | 0.25 | 0.42 | <.0001 |
| **Race/Ethnicity** |  |  |  |  |  |
| Non-Hispanic White (Referent) | 374/47227 | 1.00 | - | - | - |
| Non-Hispanic Black | 72/6676 | 1.15 | 0.88 | 1.51 | 0.2944 |
| Hispanic | 152/13268 | 1.20 | 0.97 | 1.49 | 0.0982 |
| Other | 13/1561 | 1.07 | 0.62 | 1.87 | 0.7999 |
| **Newly Diagnosed Diabetes** | 61/2439 | 3.67 | 2.75 | 4.91 | <.0001 |
| **Diabetes with Complications (Referent: No complications)** | 402/39418 | 1.52 | 1.26 | 1.82 | <.0001 |
| **Medicare Original Entitlement: Disabled (Referent: Not disabled)** | 61/5704 | 0.96 | 0.72 | 1.28 | 0.7895 |
| **Dual Eligibility (Referent: No dual eligibility)** | 137/11990 | 1.30 | 1.04 | 1.62 | 0.0235 |
| **Quartiles of Percent Living in Regions with < 12 Years Education** |  |  |  |  |  |
| [0-11] (Referent) (Very good education) | 126/14666 | 1.00 | - | - | - |
| [12-20] (Good education) | 129/15741 | 0.96 | 0.75 | 1.23 | 0.7647 |
| [21-29] (Poor education) | 158/17533 | 0.99 | 0.77 | 1.28 | 0.9472 |
| [30-100] (Very poor education) | 167/17005 | 1.02 | 0.79 | 1.31 | 0.8854 |
| **Metropolitan Areas (Referent: Non-Metro)** | 471/54367 | 0.90 | 0.73 | 1.11 | 0.3195 |
| **Elixhauser comorbidity index** |  |  |  |  |  |
| [0-1] (Referent) | 125/13538 | 1.00 | - | - | - |
| [2-3] | 213/23124 | 1.08 | 0.86 | 1.36 | 0.5151 |
| [>3] | 273/32070 | 1.01 | 0.81 | 1.26 | 0.9357 |

*OR= Odds Ratio.
