# Supplementary material for: Utilization of diabetes self-management program among breast, prostate, and colorectal cancer survivors: Using 2006–2019 Texas Medicare data
Source: PLoS One. 2023 Jul 27;18(7):e0289268. doi: 10.1371/journal.pone.0289268 (PMC10374119; doi:10.1371/journal.pone.0289268)
Supplement: Attachment — Submitted filename: Response to Reviewer.docx [file pone.0289268.s002.docx]

**Response to Reviewer Comments**

**Manuscript ID:** PONE-D-23-10647

**Manuscript Title**: Utilization of Diabetes Self-Management Program among Breast, Prostate, and Colorectal Cancer Survivors: Using 2006-2019 Texas Medicare Data

**Comments to the Author**

1. Is the manuscript technically sound, and do the data support the conclusions?

The manuscript must describe a technically sound piece of scientific research with data that supports the conclusions. Experiments must have been conducted rigorously, with appropriate controls, replication, and sample sizes. The conclusions must be drawn appropriately based on the data presented.

Reviewer #1: Yes

2. Has the statistical analysis been performed appropriately and rigorously?

Reviewer #1: Yes

3. Have the authors made all data underlying the findings in their manuscript fully available?

The [PLOS Data policy](https://nam11.safelinks.protection.outlook.com/?url=http%3A%2F%2Fwww.plosone.org%2Fstatic%2Fpolicies.action%23sharing&data=05%7C01%7Cweilee%40utmb.edu%7C0d038685baaf408a2adf08db71ff10e1%7C7bef256d85db4526a72d31aea2546852%7C0%7C0%7C638229112678976271%7CUnknown%7CTWFpbGZsb3d8eyJWIjoiMC4wLjAwMDAiLCJQIjoiV2luMzIiLCJBTiI6Ik1haWwiLCJXVCI6Mn0%3D%7C3000%7C%7C%7C&sdata=b%2BuUNXl64WLlqrJAs7Nu%2Fq%2Fx0RxYdSEsUDLuTO%2F9%2FaY%3D&reserved=0) requires authors to make all data underlying the findings described in their manuscript fully available without restriction, with rare exception (please refer to the Data Availability Statement in the manuscript PDF file). The data should be provided as part of the manuscript or its supporting information, or deposited to a public repository. For example, in addition to summary statistics, the data points behind means, medians and variance measures should be available. If there are restrictions on publicly sharing data—e.g. participant privacy or use of data from a third party—those must be specified.

Reviewer #1: Yes

4. Is the manuscript presented in an intelligible fashion and written in standard English?

PLOS ONE does not copyedit accepted manuscripts, so the language in submitted articles must be clear, correct, and unambiguous. Any typographical or grammatical errors should be corrected at revision, so please note any specific errors here.

Reviewer #1: Yes

5. Review Comments to the Author

Please use the space provided to explain your answers to the questions above. You may also include additional comments for the author, including concerns about dual publication, research ethics, or publication ethics. (Please upload your review as an attachment if it exceeds 20,000 characters)

Reviewer #1: Thanks for the opportunity to review this manuscript. The present analysis is a retrospective study of DSMT utilization among breast, prostate, and colorectal cancer survivors and associated patients’ characteristics in Texas using the registry-claims linkage data. The findings and the message of this study are valuable and informative to the journal audience. However, there are some concerns regarding the measurement of variables and delivery of findings. These concerns and other comments are listed below.

**Reply:** We are delighted to learn that the reviewer found our study valuable and informative to the journal audience. We have addressed the concerns about the literature and definitions of variables. We also expanded our discussion section, and all changes could be found in the revised manuscript. Please do not hesitate to let us know if further revision is needed to enhance the quality of our manuscript. Thank you.

Introduction
• Diabetes is a board topic. As the study is focusing on cancer survivors, the authors should include evidence related to diabetes more specific to cancer patients other than the general population to elaborate why this study is significant. For example, what is the prevalence/incidence of diabetes among the cancer population? Will the survivorship be affected by diabetes and diabetes complications? Does diabetes management improve the quality of life for cancer survivors?...
• I would suggest describing more details regarding the services and provider information of the DSMT program.

**Reply:** We thank the reviewer for your thoughtful comments. We have expanded our Introduction section to provide the audience more information related to cancer survivors, impacts of having diabetes, and DSMT programs.

Methods
• “In 2021, the TCR joined the SEER program.” is unrelated information which can be deleted.
• Were patients who had severe renal diseases or kidney transplantation excluded?
• How was dual eligibility imputed before 2007 in the regression analysis?
• What was the data source for the zip code level data (education & income)?
• Poverty level was included in the descriptive analysis but not introduced in the “Independent Variable & Covariates” section.
• How was metro/non-metro classified?

**Reply:** Thank you for your constructive feedback. We have removed the unrelated information and added more details for the study sample and variables.

Results
• Please use 1000 separator in the text and tables to be more reader friendly.

**Reply:** Thank you very much for your advice. We have applied “comma” to all tables and descriptions in the Results section.

Discussion
• The statement “the number of cancer survivors has increased almost five-fold and the number of first-time DSMES users increased almost eight-fold” is problematic. The denominators are different. For instance, the 2006 cohort is for cancer patients diagnosed in 1999-2001 and the 2019 cohort is for 1999-2014. The numbers are not comparable.
• Please consider discussing the determinants of average time of DSMT use.

**Reply:** We appreciate the insightful comment. In our Discussion section, we have added more information on the utilization and determinants of use.

6. PLOS authors have the option to publish the peer review history of their article ([what does this mean?](https://nam11.safelinks.protection.outlook.com/?url=https%3A%2F%2Fjournals.plos.org%2Fplosone%2Fs%2Feditorial-and-peer-review-process%23loc-peer-review-history&data=05%7C01%7Cweilee%40utmb.edu%7C0d038685baaf408a2adf08db71ff10e1%7C7bef256d85db4526a72d31aea2546852%7C0%7C0%7C638229112679132515%7CUnknown%7CTWFpbGZsb3d8eyJWIjoiMC4wLjAwMDAiLCJQIjoiV2luMzIiLCJBTiI6Ik1haWwiLCJXVCI6Mn0%3D%7C3000%7C%7C%7C&sdata=my97JwR9pA6lSfzy9P8cA1t%2Bjy%2Bu5TAu5zQEqt%2BLL24%3D&reserved=0)). If published, this will include your full peer review and any attached files.
If you choose “no”, your identity will remain anonymous but your review may still be made public.

Do you want your identity to be public for this peer review? For information about this choice, including consent withdrawal, please see our [Privacy Policy](https://nam11.safelinks.protection.outlook.com/?url=https%3A%2F%2Fwww.plos.org%2Fprivacy-policy&data=05%7C01%7Cweilee%40utmb.edu%7C0d038685baaf408a2adf08db71ff10e1%7C7bef256d85db4526a72d31aea2546852%7C0%7C0%7C638229112679132515%7CUnknown%7CTWFpbGZsb3d8eyJWIjoiMC4wLjAwMDAiLCJQIjoiV2luMzIiLCJBTiI6Ik1haWwiLCJXVCI6Mn0%3D%7C3000%7C%7C%7C&sdata=btDYlyysS5UtxSbL%2BPaq8IhZZkXwros4KsIUhbaAQvw%3D&reserved=0).

Reviewer #1: No
